# Trialling the SmartWorm® application in New Zealand sheep farms

**DOI:** 10.1016/j.ijpddr.2025.100616

**Published:** 2025-09-24

**Authors:** C.L. Brosnahan, D. Warburton, N. Cotter, J.C. Tanner, A.W. Greer

**Affiliations:** a Beef + Lamb New Zealand, Level 4, 154 Featherston Street, Wellington, New Zealand; b Vet Services Hawke's Bay, 801 Heretaunga Street West, Hastings, New Zealand; c Cotter Agritech, Dromtrasna North, Abberyfeale, Co. Limerick, Ireland; d Lincoln University, P.O. Box 85084, Christchurch, New Zealand

**Keywords:** Parasites, Targeted selective treatment, Anthelmintics, Gastrointestinal nematodes

## Abstract

Gastrointestinal nematodes (GIN) remain a major health and productivity challenge for grazing livestock globally, including New Zealand where widespread anthelmintic resistance has been reported. This was a pilot study evaluating the effectiveness of SmartWorm®, an app-based decision-support tool for Targeted Selective Treatment (TST) of internal parasites to reduce drench use without compromising lamb growth under New Zealand conditions.

A total of 1738 ewe lambs across three commercial farms were allocated to either a TST or Blanket Treatment (BT) group (treated every 28 days) and monitored over a 90-day period. All animals were drenched at the start of the trial, after which BT animals received treatment at each subsequent weighing. SmartWorm was used to determine drenching need for TST animals based on individual animal performance relative to expectation. Faecal egg counts (FEC), weight gain, and treatment frequency were assessed.

Across all farms, TST reduced anthelmintic use by 37–57 % compared with BT, with no significant differences in liveweight gain (P = 0.510). There was a weak but significant treatment effect on FEC (P = 0.01), and a linear relationship (R^2^ = 0.8951, P < 0.001 with one outlier removed) between BT group FEC and TST rate, indicating the system's responsiveness to parasite challenge.

This study demonstrates that implementing TST using this app can enable reduced anthelmintic use without compromising performance—an important step towards sustainable parasite management on New Zealand sheep farms.

## Highlights

• Smartworm® reduced anthelmintic use by 37–57 % without compromising liveweight gain.
• The algorithm demonstrated high predictive accuracy for drenching need.
• SmartWorm® proved to be a practical tool for Targeted Selective Treatment.

## Graphical abstract

**Figure:**
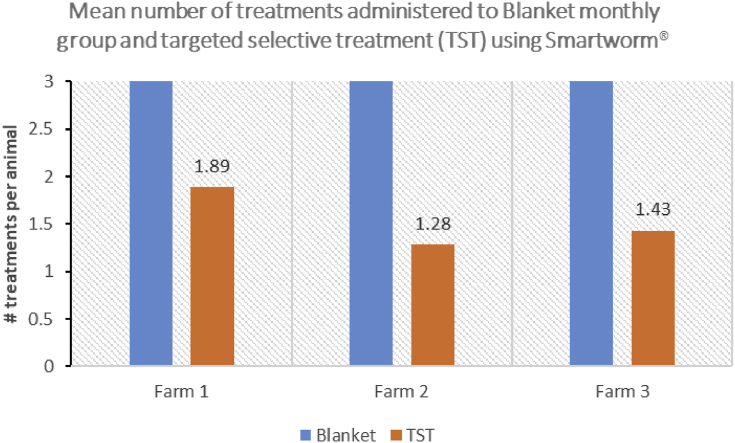

## 1 Introduction

Gastrointestinal nematodes (GIN) are a priority animal health issue for livestock farmers with management of GIN an important animal health issue for grazing ruminants globally. The use of anthelmintics for the control of parasites has resulted in resistance on numerous livestock farms globally and in New Zealand with this first reported in 1979 (Vlassoff and Kettle, 1980). A 2024 report from samples assessed at one of New Zealand's largest laboratories has shown that 51 % of farms tested had resistance to *Trichostrongylus* species with a triple-active combination anthelmintic (Riddy, 2024). While many farms have a drench resistance issue, there will still be some drenches that are effective and these need to be preserved for as long as possible.

To farm productively in the face of drench resistance requires a parasite management plan that utilises multiple tools at a farm systems level to be successful and sustainable. Anthelmintics, or drenches, should be used in combination with other approaches including the use of forages, pasture management, resistant or resilient genetics, cross-grazing and managing refugia. Targeted Selective Treatment (TST) is a refugia strategy where animals are drenched on an individual basis. TST has been shown to successfully reduce the number of drenches without compromising production while also slowing the reduction in drench efficacy (Kenyon et al., 2009). The use of TST in conjunction with the Happy Factor™ method (Greer et al., 2009) has been used in multiple studies to provide a rapid and reliable method of identifying those individuals requiring a drench (Greer et al., 2010; Kenyon et al., 2013; Busin et al., 2014; McBean et al., 2016, 2021).

SmartWorm® is a mobile app developed in Ireland by Cotter Agritech. This app builds upon the Happy Factor™ concept to determine the animals that need a drench. In conjunction with an electronic ID (eID) tag, liveweight gain and other relevant information about the farm, the app can indirectly identify animals most likely to require a dose of anthelmintic.

To date, there have been no practical commercial tools in New Zealand to automatically identify which individual animals within a mob require a dose of anthelmintic when lambs are treated for worm burden. To help farmers preserve the efficacy of drenches and reduce the amount of anthelmintics used, SmartWorm® was trialled to determine if it was appropriate for the New Zealand situation as a tool for farmers to consider in their parasite management plan for lambs. Of particular interest was whether Smartworm could be used in a practical farm setting to reduce anthelmintic input while protecting lamb liveweight gain.

## 2 Material and methods

### 2.1 Trial site and selection

Three commercial farms in the Wairarapa and Hawke's Bay region were selected for inclusion in this pilot study based on access to appropriate facilities for weighing, drafting and electronic identification (eID) over the 90-day study, flexibility to alter weighing schedules if the study required it, and a known drench efficacy for the planned active of at least 95 % by undifferentiated egg count and larval species conducted in the month prior to the study. For each farm, the SmartWorm® app was installed on an Android device and linked via Bluetooth to the eID panel or wand reader.

### 2.2 Animals and treatments

A total of 1738 ewe lamb replacements, approximately 9 months old at the start of the trial, were used across the three farms; 512 on Farm 1, 694 on Farm 2 and 532 on Farm 3. The study was carried out from the first of May until the thirtieth of August 2023.

Four visits (V1 to V4) were conducted during the study period per farm. At the first visit, all animals were yarded, weighed and drenched with an anthelmintic product known to be effective for that property to remove existing worm burdens and ensuring all farms and animals had a comparable starting infection level. The anthelmintic product differed for each farm and was dependent on them having a faecal egg count reduction test carried out in the last three years showing that drench was >95 % effective against each major species of gastrointestinal nematode present or a FEC carried out 7–10 days after the first drench was administered (post drench check) to ensure the drench was effective. Farm 1 and 3 had a full FECRT showing susceptibility to the anthelmintic used. Farm 2 did not have a FECRT as it was a new lease and timing did not allow one to be carried out before the trial started. The-post drench check was 0 epg for Farm 1 and 3 and 5 epg for Farm 2. Farm 1 and 3 used a combination containing Derquantel and Abamectin, Farm 2 used a combination containing Abamectin, Oxfendazole and Levamisole. WAAVP guidelines for FECRT (Kaplan et al., 2023)) were not strictly followed as the primary purpose of the drench check was not to determine the presence of anthelmintic resistance, but rather was to ensure a drench efficacy of the product being used on each farm above levels that may be expected to impact on productivity (McBean et al., 2021). Animals were then assigned into one of two treatment groups based on weight taken at the first day of the trial (V1) so the average group weight was similar for both treatment groups within each farm. The two treatment groups were TST Treatment (TST) group, and Blanket Treatment (BT) group. On each farm, the TST and BT groups were run together as a single mob.

Visits 2–4 occurred at 28-day intervals. At each visit animals were yarded, weighed and the SmartWorm® app used via the automated drafter to sort the animals into three groups; the BT group, the TST - no drench group (TST-ND) that required no drench and the TST-drenched group (TST-D) that required a drench. For TST animals, the app determined the treatment protocol (drench or not drench) at each weighing session based on their individualised performance target which was set to a treatment threshold of a worm rating (WR) value of 7. This was calculated through the app following farmer input of aspects of average pasture supply and quality over the preceding period. BT animals were drenched at each weighing session.

### 2.3 SmartWorm® app

The SmartWorm® algorithms integrate farm, mob, and individual-level inputs to calculate an expected growth rate for each lamb under prevailing conditions on a specific farm. This expected performance is then compared with the observed performance since the previous weighing, and the divergence is expressed as a Worm Rating (WR) value. Animals with a WR value above 7 are recommended as not requiring treatment, since they are highly unlikely to have a production benefit from it. Likewise, animals with a WR value below 7 are classified as highly likely to benefit from treatment. The recommendation is delivered in real time via a red (drench) or green (no drench) light on the mobile app 'sheep side', and the system can also instruct auto drafting equipment to rapidly separate dose and no dose animals. SmartWorm® randomly selects a small ‘control group’ of blanket-treated individuals in every mob, providing a performance baseline to compare against the TST portion and giving farmers confidence in the approach. In the current study, the size of the 'control group' was set at 50 % of the mob and was manually selected on each farm to be balanced for starting weight as explained earlier in this paper.

The inputs to the algorithm include individual liveweight and liveweight gain since the previous weighing, pasture availability and quality, preceding 28-day and forecasted 10-day rainfall and temperature data (sourced from the national New Zealand weather database and forecasting models of www.metservice.com as local to the farm location as possible).

### 2.4 Parasitology

At each visit, 15 fresh faecal samples were taken from the ground of each of treatment groups, *viz* BT and TST groups at visit 1 (n = 30) and in subsequent visits each of BT, TST-ND and TST-D groups (n = 45). Samples were taken immediately after being voided into separate containers which were then sealed prior to transport via courier as soon as was practically possible to the testing laboratory. Faecal Egg Counts were conducted on individual faecal samples by Awanui Veterinary using a modified McMaster method with a sensitivity of 50 eggs per gram. A larval culture was carried out as a pool from each group by Awanui Veterinary. Faecal pools were incubated at 27 °C ± 2 °C for seven days. Identification of 3rd stage larvae was carried out as per the Australia and New Zealand Standard Diagnostic Procedures (Hutchinson, 2009).

To investigate lower than expected animal growth rates on the farm, pasture analysis was conducted prior to visit 3 and sent to Hill Labs, New Zealand, for energy, protein and dry matter analysis. Trace mineral analysis of the animals via blood sampling was also carried out on Farms 1 and 2 prior to visit 3. Vitamin B12 and selenium levels were tested by Awanui Veterinary, New Zealand. All analysis was shown to be within the suggested reference ranges (Supplementary Table 1).

### 2.5 Data analysis and dataset cleaning

Animal ID, live weight, worm rating (WR) value, number and timing of treatments from all properties was provided from the SmartWorm® application for analysis. Not all animals were present at each sampling time, the reasons for which were not able to be determined as no animal deaths were reported by the farmers. For V2, V3 and V4, respectively, Farm 1 had 487, 433 and 494 animals present, Farm 2 had 680, 676 and 652 animals present, and Farm 3 had 503, 496 and 453 animals present. Due to the impact a missing animal live weight data point has on the calculation of WR both at that time and subsequent weighing events and the impact on the calculation of the response to treatment, data was cleaned to allow only animals with full sets of data to be included in the analysis. Farm 1 was reduced to 383 animals, 185 in the BT group and 198 from the TST group. Farm 2 was reduced to 612 animals, 298 from the BT and 314 from the TST group. Farm 3 was reduced to 419, 208 from the BT and 211 from the TST group. All animals were drenched at visit 1, so this data was not included in the analysis.

### 2.6 Statistical analysis

Analysis was initially carried out using MS Excel (Microsoft Corporation, 2024). A receiver Operator Characteristic (ROC) analysis was carried out using STATA 17 (StataCorp LLC, 2021; College Station, TX, U.S.A.) based on the change in WR for all treatment animals calculated by the app in the month after treatment relative to the Worm Rating at the time of treatment to determine if the threshold set for this study was appropriate for each farm. The optimum WR a compromise between true positives, being those that had a WR of less than 7 and whose WR increased following treatment, and false positives, being those that had a WR of less than 7 but whose WR did not increase following treatment. This was determined from the ROC analysis using the maximum value of sensitivity (Sn) plus specificity (Sp) where Sn = True positives(TP)/(TP + False negatives (FN)) and Sp = True negatives (TN)/(TN + False positives (FP).

Combined farm analysis was carried out using Genstat 24 (Twenty-fourth Edition, version 24.1.0.1041, VSN International Limited, United Kingdom). Live weight was analysed with repeated measures using restricted maximum likelihood analysis (REML) after undergoing sequential comparison of ante-dependence structures. Cumulative liveweight gain, starting live weight and the mean number of drenches administered per animal were analysed using unbalanced ANOVA. For cumulative liveweight gain and starting live weight, comparisons were made with the individual animal as a replicate both within farm and then overall when blocked for farm with post-hoc comparisons made using a Fishers Unprotected LSD.

Log10(n+1) FEC were analysed using an unbalanced design ANOVA, with treatment, time, and farm as factors. Time and farm were found to have a significant effect (P < 0.001 for both) and data was subsequently re-analysed using a Restricted Maximum Likelihood Analysis (REML) blocked for farm. The relationship between the mob average FEC for the blanket group and the proportion of TST animals treated was analysed using simple linear regression.

## 3 Results

### 3.1 Parasitology

Mean group FEC at each time and larval cultures at visits 1 to 4 are shown in Table 1. Initial FEC from a bulk sample was moderate to high for Farm 1, being 2493 epg at V1, around 2000 epg for all groups at V2 and then declining to low-moderate levels at V3 and V4. For Farm 2 FEC at V1 were just 3 epg which then increased in all groups at subsequent time points but remained below 200 epg throughout. Samples for FEC were not available from Farm 3 on V 1 and V2 but at V3 and V4 were at low to moderate levels of between 157 and 623 epg for all groups. This aside, both time and farm were found to have a significant effect (P < 0.001 for both) and data was subsequently re-analysed using a Restricted Maximum Likelihood Analysis (REML) blocked for farm. Overall, there was an effect of treatment group on FEC (P = 0.01) and a trend for a treatment × time interaction (P = 0.095) reflecting slightly lower FEC for the TST-D group on two of the four sampling times but a similar or greater FEC for this group at V4 across all farms.

**Table 1:** Arithmetic mean FEC (eggs per g of faeces) and % species from copro-culture results for Blanket (BT), TST-drenched (TST-D) and TST not drenched (TST-ND) at each sampling time. Mob samples taken from a random sample at each time, not repeated measures of the same individuals.

		Initial FEC	BT	TST-D	TST-ND	*Haemonchus contortus*	*Teladorsagia circumcincta*	*Trichostrongylus* spp.	*Cooperia* spp.	Oesophagostomum/Chabertia
**Farm 1**	V1	2493				40	4	32	24	0
	V2		2267	1733	1963	–	–	–	–	–
	V3		763	463	1053	0	6	94	0	0
	V4		243	790	353	–	–	–	–	–
**Farm 2**	V1	3				0	30	54	16	0
	V2		113	67	142	–	–	–	–	–
	V3		50	146	113	0	12	46	18	24
	V4		13	177	143	–	–	–	–	–
**Farm 3**	V1	–				–	–	–	–	–
	V2		–	–	–	–	–	–	–	–
	V3		253	157	623	0	16	84	0	0
	V4		277	627	347	–	–	–	–	–

Coproculture results revealed that *Haemonchus contortus* was detected for Farm 1 at V1 only, being 40 % of the culture. Thereafter, *Trichostrongylus* spp. were dominant. For Farm 2 *circa* 50 % *were Trichostrongylus* spp. with the remainder split between *Teladorsagia circumcincta* and *Cooperia* spp. For farm 3 there was just one culture result with *Trichostrongylus* spp. comprising 84 % of the population.

### 3.2 Anthelmintic treatments

**Fig. 1:**
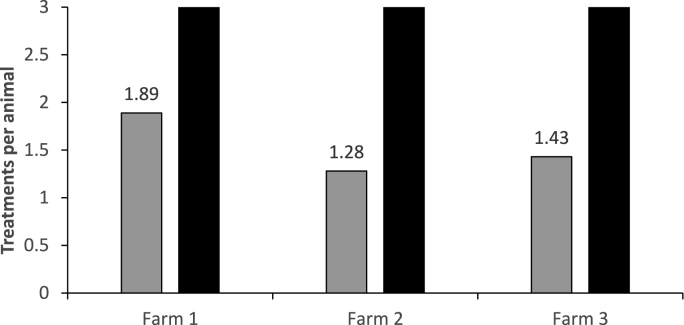
The mean number of treatments administered to animals using the Smartworm algorithm (TST: grey bars) and monthly blanket treated (BT: solid bars) treatments for each farm.

The number of treatments administered at each visit (V2 to V4) for each farm and total number of treatments administered to TST animals for each farm is shown in Table 2 and Fig. 1, respectively. For Farm 1, the TST group had a total of 374 drenches administered, being an average of 1.89 drenches per animal compared with 3 drenches administered to each of the animals in the BT group. After the first drench at V1, one TST animal didn't require any further treatment, 35 required one further drench, 147 required two more drenches and 15 required drenching at all three times. Of the drenches given to the TST animals a majority of these were administered on V2 and V3. Of the 147 administered on V2 118 animals required a further drench on V3.

**Table 2:** Number of drenches given at each farm visit (V2-V4) and total drenches for animals in each treatment group, BT = blanket treatment, TST = Targeted Selective Treatment for each farm.

	Group	No of Drenches	No of Animals	Drenched V2	Drenched V3	Drenched V4	Total Drenches
**Farm 1**	**Blanket**	3	**185**	**185**	**185**	**185**	**555**
	**TST**			**196**	**133**	**45**	**374**
		0	1	0	0	0	0
		1	35	34	0	1	35
		2	147	147	118	29	294
		3	15	15	15	15	45
**Farm 2**	**Blanket**	3	**298**	**298**	**298**	**298**	**894**
	**TST**			**311**	**62**	**29**	**402**
		0	1	0	0	0	0
		1	229	227	2	0	229
		2	79	79	55	24	158
		3	5	5	5	5	15
**Farm 3**	**Blanket**	3	**208**	**208**	**208**	**208**	**624**
	**TST**			**211**	**59**	**32**	**302**
		0	0	0	0	0	0
		1	144	144	0	0	144
		2	43	43	35	8	86
		3	24	24	24	24	72

For Farm 2, 402 drenches were administered to the TST group, being an average of 1.28 drenches per animal with three drenches administered to each of the animals in the BT group. Of the drenches given to the TST animals a majority of these (311) were administered on V2. Of the 311 administered on V2, 227 animals did not receive any further drenches. For 79 of the animals treated on V2, 55 also received a drench at V3 while 24 received their only other drench on V4. Five animals needed drenching on each occasion. One animal did not require any further drenches after V1.

For Farm 3 there was a total of 302 drenches given to the TST group, an average of 1.43 drenches per animal compared with 3 drenches administered to each of the animals in the BT group. Of the drenches given to the TST animals a majority of these were administered on V2 when all animals were drenched due to technology issues at this farm on at this sampling time. One-hundred and forty-four TST animals received a treatment at V2 only and did not require further treatment, 43 required drenching twice and 24 required drenching all three times.

**Fig. 2:**
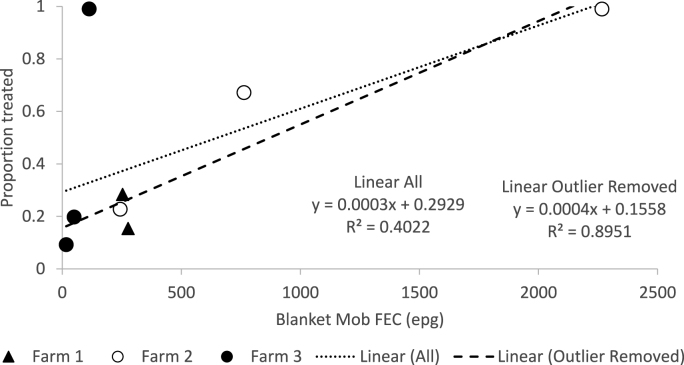
Relationship between mob average FEC for the blanket treatment group, as a proxy for worm challenge since previous treatment and the proportion of TST animals treated with all farm data included and when one ‘outlier’ value for farm 3 was excluded. Intercept of the line of best fit (linear) represents the proportion that would be treated when FEC = 0.

The relationship between the proportion of TST animals treated and the mob average FEC for the BT group and is given in Fig. 2. A linear correlation was shown with increasing proportion treated as FEC increased. With all data together the equation of best fit was y = 0.000317*x* + 0.293 (R^2^ = was 0.4022, P = 0.09) and with the one outlier from Farm 3 when all animals were treated due to technological issues was removed the best fit linear equation was y = 0.0003941*x* + 0.1558 (R^2^ = 0.8951, P = 0.001), indicating that even with a 0 epg, 15.6 % of lambs would be expected to be treated.

### 3.3 Animal performance

The starting live weights and cumulative liveweight gain (LWG) of the TST and BT groups, and for the TST animals relative to the number of treatments received are shown in Table 3. Overall and for each of Farms 1, 2 and 3 liveweight gain between TST and BT was similar (P > 0.05 for all). Cumulative LWG was not different, being 0.5 kg (P = 0.07) and 0.2 kg (P = 0.51) lower on average in TST compared with BT in Farms 1 and 3 and 0.2 kg greater (P = 0.18) in Farm 2. For each farm and overall, the cumulative LWG in TST animals decreased with increasing number of treatments received. Combining all three farms data over the three months of the trial, for live weight there was an effect of time (P < 0.001) but there was no effect of treatment on liveweight gain (P = 0.964) or treatment x time interaction (P = 0.837), reflecting an increase in live weight with time that was not different for TST or BT lambs. With farm included as a factor there was a treatment x time x farm interaction for live weight (P = 0.04) reflecting similar liveweight across all time points for both treatments within each farm but final live weights being 0.26 kg heavier for TST compared with BT for Farm 2 but lighter by 0.29 kg and 0.43 kg for Farm 3 and Farm 1, respectively. Total cumulative LWG showed cumulative LWG was not affected by treatment (P = 0.510), being 6.69±0.099 kg and 6.60±0.097 kg for BT and TST, respectively. For each farm and overall, it can be observed the starting live weight was generally heavier for those that received more treatments (Table 3).

**Table 3:** Mean (±s.e.m) starting live weight (kg) and cumulative liveweight gain (LWG) for blanket monthly treated (BT) and targeted selective treatment (TST) animals in total and for the number of treatments administered to each of the TST animals. Data is for each farm and overall, with all farms combined. For comparisons between number of treatments administered within the TST treatment, different superscripts indicate significant differences (P < 0.05).

Treatment	BT	TST Total			TST		
No of drenches	3			0	1	2	3
**Farm 1**							
n	185	198		1	35	147	15
Starting LW (kg)	34.1 ± 0.26	34.2 ± 0.25	*P=0.78*	28.0	33.3 ± 0.57^ab^	34.3 ± 0.28^ab^	35.2 ± 0.84^b^
LWG (kg)	8.9 ± 0.21	8.4 ± 0.19	*P=0.07*	7.5	10.7 ± 0.30^b^	8.4 ± 0.19^b^	3.5 ± 0.48^a^
**Farm 2**							
n	298	314		1	229	79	5
Starting LW (kg)	37.4 ± 0.23	37.5 ± 0.21	*P=0.98*	31.0	36.9 ± 0.13^a^	39.1 ± 0.21^b^	37.6 ± 0.59^ab^
LWG (kg)	5.4 ± 0.14	5.6 ± 0.12	*P=0.18*	10.0	6.2 ± 0.13^b^	4.0 ± 0.21^a^	2.7 ± 0.59^a^
**Farm 3**							
n	208	211		–	144	43	24
Starting LW (kg)	40.6 ± 0.21	40.5 ± 0.22	*P=0.71*	–	39.7 ± 0.25^a^	41.2 ± 0.42^b^	43.5 ± 0.54^c^
LWG (kg)	6.6 ± 0.18	6.4 ± 0.21	*P=0.51*	–	7.5 ± 0.19^c^	4.7 ± 0.60^b^	2.6 ± 0.20^a^

**Overall**							
n	691	723		2	408	269	44
Starting LW (kg)	37.5 ± 0.16	37.4 ± 0.16	*P=0.82*	29.5 ± 1.06^a^	37.6 ± 0.19^c^	36.8 ± 0.27^b^	40.0 ± 0.75^d^
LWG (kg)	6.7 ± 0.11	6.6 ± 0.11	*P=0.60*	8.75 ± 0.88^b^	7.1 ± 0.12^c^	6.5 ± 0.20^b^	2.9 ± 0.22^a^

For each farm, the response to treatment as determined by the ROC analysis of the change in WR values gave an area under the curve of 0.8658, 0.9130 and 0.9025 for Farms 1, 2 and 3 respectively, and with all data combined (Supplementary Fig. 1) an area under the curve of 0.8957 indicating very good to excellent differentiation between true positives and false positives. For all data combined the maximum value of Sn + Sp was 1.63 from a sensitivity of 0.85 and a specificity of 0.78, which occurred with a treatment threshold WR of 7.0.

## 4 Discussion

The ability of this TST enabling app to reduce drench in the New Zealand context for this age group of lambs was demonstrated. There was a reduction of drench compared with blanket treatment lambs of 49 % on average. Given the FEC of the TST-ND animals were generally comparable with TST-D animals, this presumably would have provided considerable refugia within the parasite population. Additionally, a significant cost savings to each business of the drench product, labour and helping preserve the current drenches (Kenyon et al., 2013) to avoid use of the more expensive novel acting drenches and potential environmental benefits (Sands and Knoll, 2021) with minimal impact on average lamb liveweight gain. However, this study is not without its limitations that must be considered. While the intention of this pilot study to provide an initial evaluation of the utility of the Smartworm® App was achieved, it involved a small number of farms. As expected, these farms varied in both the likely parasite challenge level and species present. Despite this, the outcome of reduced drench use with little impact on lamb performance was consistent across all farms, which is in line with the previous investigations in the United Kingdom which reported consistency of a similar approach across farms (McBean et al., 2021). Further, with the limited time frame late in the grazing season, caution must be applied if extending the current results to lambs of all ages across different environments. In this trial, faecal egg count was not taken into consideration as part of the app. The 2025 version of the app now includes pre-treatment mob faecal egg count and larval culture results where available to account for *Haemonchus contortus* challenge.

Overall, the results of this trial suggest that the TST regime did not impact animal performance. The cumulative liveweight gain for TST and BT animals was similar for each property and overall differed by just 0.1 kg despite nearly half the number of treatments given to TST animals. In fact, animal performance was inversely proportional to the number of treatments given, with lesser cumulative liveweight gain apparent in those that received more treatments. This was expected due to the nature of the treatment selection criteria whereby slower growing animals are more likely to be identified as requiring drenching. Interestingly, initial live weight also appeared to be associated with the number of treatments, with more treatments administered to those that started at a heavier weight. This is in line with observations of Keegan et al. (2018) who reported the heaviest animals did not show greater resilience than lighter animals when treatment was withheld. On the one hand this may reflect the animals stage of development, and an increased perceived need for treatment as they reach a point in their relative maturity where they encounter the nutritional costs associated with the acquisition of immunity (Kimambo et al., 1988; Greer et al., 2005; Dever et al., 2016; Greer and Hamie, 2016; Douhard et al., 2025). However, if this was the case it may also be anticipated that the FEC of those that needed drenching (TST-D) might be lower than those not drenched (TST-ND), which was not consistently observed. Alternatively, the greater starting live weight of those animals that received more treatments may reflect an inherent bias within the app. It may be possible the expected growth rate and/or growth potential of heavier animals are over-estimated, leading to a greater number failing to reach their target level of performance. This does highlight one of the obvious restrictions of a TST regime based on animal performance whereby it is not definitive that the cause of poorer than expected performance is due to parasitism. In the current study the proportion of TST animals treated at each time was positively correlated with mean FEC of the BT group (Fig. 2). Given the FEC of BT animals is likely to reflect the level of challenge since the last treatment, this does provide support that parasitism was a contributing factor to the perceived need for treatment of individuals. With that in mind, given that overall animal performance was not different despite a considerable reduction in drench use, this study also highlights the lack of need or benefit of treating all animals at every treatment time.

It is important to consider the use of TST as part of the farm parasite management plan. Leaving an increasing proportion of animals undrenched will increase the amount of larval contamination in the pasture in the future. The current study's aim was to understand if TST was appropriate for use in a commercial farm setting and so the TST and BT groups were grazed together. Therefore, undrenched animals benefited from treatments given to other animas in the group and no effect on pasture contamination was able to be measured. Previous studies by Kenyon et al. (2013) have shown an increase in pasture contamination compared with monthly blanket treatments when using a TST that reduced drench input by a similar amount to that shown in the current study although pasture contamination was not greater than those treatments that extended drench interval beyond 4 weeks. It is likely a similar outcome may have been expected here if TST and BT animals were grazed in separate farmlets, although in slight contrast recent modelling of the full host-parasite-environment system for *T. circumcincta* has suggested refugia strategies may not necessarily increase pasture contamination (Benson et al., 2025). The premise of drenching young animals every 28 days, which is a common practice in New Zealand, was not because the animals required a drench, but so the larval contamination in Autumn was reduced (Vlassoff and Brundson, 1981). Alternative drenching strategies require understanding of any flow on impacts and where necessary mitigation strategies to be put in place. Leathwick et al. (2006) demonstrated that leaving 10 % of lambs undrenched increased the amount *Haemonchus contortus and Trichostrongylus* spp. in the pasture by 7–10 times, respectively. However, levels of other species of parasites (*Teladorsagia circumcincta, Cooperia* spp. and *Nematodirus* spp.) were not significantly different. Interestingly, the liveweight gains under the different treatments in the Leathwick et al. (2006) study was not different between treatments. These results demonstrate the complex relationship between parasite contamination, parasite species, environment, and other management practices and the need to incorporate parasite management as a whole farm system approach with regular monitoring to ensure optimal outcomes. Furthermore, parasite management practices must balance the impact of parasites on the performance of animals and the selective forces for anthelmintic resistance (Pomroy, 2006). This highlights the need for future studies to explore the epidemiological impacts when considering refugia based strategies.

Using TST via the app requires the user to input certain parameters to determine which animals are treated or not. This includes the feeding levels and pasture quality which are used to calculate the expected weight gain of each individual. The current study highlighted two points; one, that the farmers in the study overestimated how much their animals would be growing in winter and two, the variability within the mob of weights and average daily gains. This led to further investigation of the feed quality, mineral status and close examination of the larval species present, none of which could account for the lower than expected level of performance. Understanding performance at an individual level with the technology to analyse the data in real time is a significant improvement to precision agriculture. The benefit of precision livestock farming in Scottish ewe flocks has previously been the subject of investigation (Morgan-Davies et al., 2018) but few studies have considered this in a New Zealand context. Animals requiring fewer treatments in the current study exhibited tolerance while their resistance status remains unknown and may be expected to have superior genetic merit for production traits (Greer et al., 2013). The timing of need for treatment in combination with liveweight gain data, all of which is automatically collected through the app, has the potential to provide valuable awareness and insights for the farmer as to the likely causes of impaired productivity at key times in the production cycle. Ensuring we can use this new technology with other measures to benefit the farm business in ways other than just the provision of refugia needs to remain a focus to enhance farmer adoption. However, before making widespread recommendations to sheep farmers, this technology should be trialled on more farms across different environments, with younger animals that are more naïve, and in areas known to have a *H. contortus* challenge.

## 5 Conclusion

This study demonstrated that TST in New Zealand lamb production systems can be used, with an average drench reduction of 49 % compared with blanket treatment. This reduction offers significant benefits, including likely increased refugia, decreased reliance on anthelmintics, and cost savings associated with drench products, labour, and the possibility of delayed use of more expensive novel actives for general gastrointestinal parasite control. However, the use of TST must be integrated thoughtfully into whole-farm parasite management plans that take into account the potential for greater pasture larval contamination over time in balance with the risk of developing anthelmintic resistance. This underlies the need for ongoing monitoring and adaptive management strategies. Further trials are recommended across a wider range of farms, regions, and environmental conditions to fully assess the suitability and robustness of SmartWorm® as a decision-support tool for sustainable parasite control in sheep systems.

## CRediT authorship contribution statement

**C.L. Brosnahan:** Writing – review & editing, Writing – original draft, Project administration. **D. Warburton:** Writing – review & editing, Supervision, Project administration, Methodology, Investigation, Funding acquisition, Conceptualization. **N. Cotter:** Writing – review & editing, Resources. **J.C. Tanner:** Writing – review & editing, Formal analysis, Data curation. **A.W. Greer:** Writing – review & editing, Methodology, Formal analysis, Data curation.

## Ethics approval

Animal use was in accordance with animal ethics approval by the Lincoln University Animal Ethics Committee, application number LUAEC2023-19.

## Conflict of interest statement

This study was funded by Beef + Lamb New Zealand, an organisation funded by New Zealand farmers, who were involved in the writing of the manuscript; and in the decision to publish the results. The technology evaluated in this study is owned by Cotter Agritech, whose representatives reviewed the manuscript prior to submission. The authors declare no other conflicts of interest.
