# Supplementary material for: Trialling the SmartWorm® application in New Zealand sheep farms
Source: Int J Parasitol Drugs Drug Resist. 2025 Sep 24;29:100616. doi: 10.1016/j.ijpddr.2025.100616 (PMC12509729; doi:10.1016/j.ijpddr.2025.100616)
Supplement: Multimedia component 1 [file mmc1.docx]

**Supplementary Figure 1:** Combined data from all drenched animals from both BT and TST groups across all times and all farms for: (A) the response to treatment (% change in post treatment worm rating (WR)) relative to pre-treatment WR. (B) Receiver Operator Characteristic analysis of the area under the curve for sensitivity and 1 minus specificity to determine the likelihood of a positive change in WR due to treatment. (C) Additive values of sensitivity (Sn; True positives(TP)/(TP+False negatives (FN)) and specificity (Sp; True negatives (TN)/(TN+False positives (FP)) based on the likelihood of a positive response to treatment relative to the WR at the time of treatment with maximum value used to determine the optimum WR to use as a treatment threshold.
