# Supplementary material for: Trialling the SmartWorm® application in New Zealand sheep farms
Source: Int J Parasitol Drugs Drug Resist. 2025 Sep 24;29:100616. doi: 10.1016/j.ijpddr.2025.100616 (PMC12509729; doi:10.1016/j.ijpddr.2025.100616)
Supplement: Multimedia component 2 [file mmc2.docx]

**Supplementary Table 1**: Pasture quality assessment and reference ranges for the supply of metabolizable energy (MJME per kg dry matter (DM); MJME/kgDM), Crude Protein %, DM % on each farm and serum vitamin B12 and selenium (Se) from lambs on Farms 1 and 2.
